# Mitochondrial a*tp9* genes from petaloid male-sterile and male-fertile carrots differ in their status of heteroplasmy, recombination involvement, post-transcriptional processing as well as accumulation of RNA and protein product

**DOI:** 10.1007/s00122-014-2331-x

**Published:** 2014-06-10

**Authors:** Marek Szklarczyk, Mateusz Szymański, Magdalena Wójcik-Jagła, Philipp W. Simon, Andreas Weihe, Thomas Börner

**Affiliations:** 1 Unit of Genetics, Plant Breeding and Seed Science, Institute of Plant Biology and Biotechnology, Faculty of Horticulture, University of Agriculture in Krakow, Al. 29 Listopada 54, 31-425 Kraków, Poland; 2 USDA, Agricultural Research Service, Vegetable Crops Research Unit, Department of Horticulture, University of Wisconsin, 1575 Linden Drive, Madison, WI 53706 USA; 3 Institute of Biology, Humboldt University, Chausseestr. 117, 10115 Berlin, Germany

## Abstract

*****Key message***:**

**Petaloid cytoplasmic male-sterile carrots exhibit overexpression of the mitochondrial** ***atp9*** **genes which is associated with specific features in organization and expression of these sequences.**

**Abstract:**

In carrots, the S_p_-cytoplasm causes transformation of stamens into petal-like organs, while plants carrying normal N-cytoplasm exhibit normal flower morphology. Our work was aimed at characterization of distinct features both cytoplasms display with respect to organization and expression of the mitochondrial *atp9* genes. We show that two carrot *atp9* genes, previously reported as cytoplasm-specific, in fact occur in heteroplasmic condition. In the S_p_-cytoplasm the *atp9*-*1* version dominates over *atp9*-*3*, while in N-cytoplasmic plants this proportion is reversed. Herein, we also indicate the presence and recombination activity of a 130-/172-bp sequence repeat which likely shaped the present organization of carrot *atp9* loci. Furthermore, cDNA sequence examination revealed that the *atp9* open reading frames (ORFs) were C to U edited in 4 nucleotide positions. One of the editing events turns a glutamine triplet into the stop codon, thereby equalizing ORFs of *atp9*-*1* and *atp9*-*3*. A certain fraction of partially edited molecules was identified—they all represented the *atp9*-*3* sequence. In either S_p_- or N-cytoplasmic plants multiple 5′ transcript termini were observed. Of these, the ones mapping more distantly from the *atp9* ORF were more pronounced in case of petaloid accessions. It was also shown that despite comparable copy number of the genomic *atp9* sequences, the level of the respective mRNAs was approximately 3 times higher in case of petaloid carrots. The latter fact corresponded to the elevated content of the ATP9 protein in plants carrying S_p_-cytoplasm. The semi-fertile phenotype of such plants is associated with a drop in ATP9 accumulation.

**Electronic supplementary material:**

The online version of this article (doi:10.1007/s00122-014-2331-x) contains supplementary material, which is available to authorized users.

## Introduction

In carrots (*Daucus carota* L.) carrying the S_p_-cytoplasm stamens are replaced with petal-like organs and thereby male reproductive function is eliminated. Such carrots represent a more general phenomenon of cytoplasmic male sterility (CMS), which refers to as maternally inherited impairment of pollen production. Petaloid male-sterile plants were found among wild carrots from USA, Canada, Sweden and Germany. The American sources of sterility were subsequently introduced into the cultivated germplasm allowing production of the modern hybrid varieties (reviewed in Bach 2000). Stamen petaloidy in cultivated carrots was also induced upon cytoplasm transfer from *D. c. maritimus* (Nothnagel et al. 1997).

Carrots representing petaloid type of CMS are not exceptional among plants. Similar abnormalities have been reported for a few other genera like *Nicotiana*, *Brassica* and *Plantago*. Most cases of CMS in tobacco resulted from interspecific hybridization and recurrent backcrossing placing *Nicotiana tabacum* nuclear genome in the context of cytoplasm from some wild *Nicotiana* species (Kaul 1988; Bonnett et al. 1991). Cytoplasm transfer in tobacco usually affects organ formation in whorls 2 and 3. Among cytoplasm donors, *N. benthamiana*, *N. bigelovii* and *N. undulata* were shown to induce transformation of stamens into petaloid structures. This feature may be accompanied by feminization of stamens, including the presence of stigmatoids. Alloplasmy was also exploited to generate male-sterile brassicas. Among those, petaloidy was reported in case of *Brassica oleracea* (broccoli) carrying *B. nigra* cytoplasm (Pearson 1972) as well as for a number of allied cytoplasms introduced into *B. juncea* (mustard) nuclear environment (Prakash 2001; Jing-Hua et al. 2005). Contrary to the situation found in *Nicotiana* and *Brassica*, petaloid CMS in *Plantago lanceolata* (called CMSII or P-type) occurs spontaneously in the field exhibiting condition known as autoplasmy (de Haan et al. 1997).

Mutations in certain nuclear genes may also condition the conversion of stamens into petals. *Agamous*—floral homeotic mutant of *Arabidopsis—*and *plena*—its counterpart from *Antirrhinum*—are by far the best studied instances of this phenomenon (Causier et al. 2010). According to the ABC(DE) model of floral morphogenesis (Ferrario et al. 2004), both mutants represent impairment of the so-called C function and its replacement with activity of genes representing A function. Joint actions of the A, B and E functions specify petal identity. Phenotypic similarities between petaloid CMS and nuclear homeotic mutations suggest that the cytoplasm, by an as yet unknown mechanism, influences the expression of transcription factors controlling floral patterning (Linke et al. 2003). It seems that cytoplasmic factors could act either through impairment of the C function or by enhancing the functions engaged in petal formation. In favor of the latter possibility, the combined ectopic expression of *AP3*, *PI* (both are B function genes) and *AP1* (A function) as well as *AP3*, *PI* together with *SEP3* (E function) converts vegetative leaves into petaloid organs (Honma and Goto 2001).

Proper elucidation of nucleo-cytoplasmic interrelations, which lead to expression of stamen petaloidy, will not be possible without prior knowledge of cytoplasmic CMS factors. These factors were identified for a number of CMS systems, and all represent mutated mitochondrial loci, in most cases formed by intragenomic sequence rearrangements (Schnable and Wise 1998; Carlsson et al. 2008). Although several reports were dedicated to characterization of such sequences in carrots and other species with petaloid type of CMS, unambiguous sterility determinants have not been indicated to date (Linke et al. 2003 and references therein). We previously described two functional carrot *atp9* genes: *atp9*-*1*—found in petaloid forms and its N-cytoplasmic counterpart—*atp9*-*3*. Moreover, a truncated *atp9* version (*atp9*-*2*) was detected in both male-fertile and male-sterile cytoplasms. This pseudogene is part of a large repeated region in the carrot mitochondrial genome (Szklarczyk et al. 2000; Iorizzo et al. 2012). We found that due to a point mutation, the open reading frame (ORF) of *atp9*-*1* is 13 amino acid residues longer than that of *atp9*-*3*. It was also shown that in petaloid accessions, *atp9* sequences were co-transcribed with the upstream *rrn5* gene and that the occasionally observed semi-fertile phenotype might be accompanied by elimination of the respective mRNA species. In the present work, specific features of carrot *atp9* sequences from the S_p_- and N-cytoplasm were further examined with a special focus on quantitative aspects of their organization and expression.

## Materials and methods

### Plant material

All studied petaloid CMS lines (A88A, 2163A, 2874A) and their respective maintainers (A88B, 2163B, 2874B) were provided by PHRO Krzeszowice (Krzeszowice, Poland). Since backcrossing of the maintainer to its male-sterile counterpart was carried for at least seven generations, a given pair of the A and B lines represents the same nuclear genotype in the context of S_p_- and N-cytoplasm, respectively.

### Isolation of nucleic acids

Total genomic DNA and total cellular RNA were isolated as described by Szklarczyk et al. (2000). Young leaves and young umbels were used for DNA and RNA extraction, respectively.

### Reverse transcription

Prior to reverse transcription, RNA samples were subjected to DNase treatment. It was carried at 37 °C for 30 min. in a 10 μl solution containing 1 μg of total cellular RNA, 1× reaction buffer (10 mM Tris–HCl pH 7.5, 2.5 mM MgCl_2_, 0,1 mM CaCl_2_), 10 U of ribonuclease inhibitor (MBI Fermentas) and 5 U of RNase-free DNase I (Boehringer Mannheim). To stop the reaction, the samples were supplemented with 1 μl of 25 mM EDTA and incubated in 65 °C for 10 min.

Resulting RNA preparation was directly used in reverse transcription set up with components and procedure of the First Strand cDNA Synthesis Kit (MBI Fermentas). This reaction was primed using either gene-specific oligo R (see below) or random hexamers from the kit. The former was used to produce cDNA for *Pfu*-driven amplification, the latter—to yield template for real-time PCR.

### DNA amplification

Details of conventional PCR are given in Szklarczyk et al. (2000).

Whenever amplification products were meant to clone for the purpose of sequence analysis, the reactions were driven with *Pfu* DNA polymerase. In this case, the reaction mixture contained: 1× PCR buffer (with MgSO_4_, MBI Fermentas), 0.2 mM dNTPs, 0.4 μM either primer, 0.05 U/μl *Pfu* DNA polymerase (MBI Fermentas) and either 0.2 ng/μl of total genomic DNA or tenfold diluted products of reverse transcription. Parameters of temperature cycling remained as in conventional PCR (Szklarczyk et al. 2000).

Real-time PCRs were prepared with the use of Smart Kit for Sybr Green I (Eurogentec). Single 25 μl reaction contained 1× Smart reaction buffer, 1 μM each primer, 66,000-fold diluted Sybr Green I stock and either 20 ng of total genomic DNA or 50-fold dilution of the reverse transcription products. The reactions were carried in triplicate in a Cepheid SmartCycler running the program: 50 °C for 2 min., 95 °C for 10 min., 35 cycles of 95 °C for 15 s and 60 °C for 1 min. (optics on), followed by a temperature ramp from 50 to 95 °C at 0.1 °C/s (optics on). Finally, the reactions were hold at 50 °C for 2 min. Quantitation was performed using comparative C_t_ (2^−ΔΔCt^) method where amount of the target sequence in line 2163A was calculated taking line 2163B as a calibrator. The C_t_ values of both lines were normalized to the sequence of actin gene amplified with primers ac-f and ac-r (see below).

### DNA cloning and sequencing

Prior to cloning, the PCR products were column-purified (Qiaquick PCR Purification Kit) and A-tailed according to the procedure given in Promega technical manual no. TM042. Ligation was performed with the use of commercially available T/A cloning kits and dedicated procedures. Ligations were transformed into DH10B *Escherichia coli* cells according to standard procedures (Promega technical manual no. TM042). White colonies were subjected to colony PCR with the insert primers in order to confirm the presence of appropriate sequences. For template preparation, colonies were picked with sterile pipette tips, suspended in 100 µl of water and then heated in boiling water bath for 10 min. After brief chilling on ice, the samples were spun at 20,000×*g* for 5 min. in 4 °C, and 1 μl of the resulting supernatant was applied in a single 10 μl reaction. The remaining reaction conditions followed those for conventional PCR (Szklarczyk et al. 2000). The positively verified clones were grown overnight in LB broth supplemented with 100 μg/ml ampicillin and then used for plasmid DNA isolation (Wizard Plus SV Minipreps DNA Purification System, Promega). Sequencing reactions were set up with the ABI Prism BigDye chemistry according to the manufacturer recommendations. The termination products were purified using ethanol/sodium acetate precipitation (Abi Prism BigDye Terminator Cycle Sequencing Ready Reaction Kit Protocol) or Sephadex pillars (https://dna.biotech.wisc.edu) and then run at a sequencing facility on Abi Prism 377 DNA sequencer.

### Primer extension analysis

Unless otherwise indicated, primer extension experiments were performed as described in Sambrook et al. (1989). The primers were labeled at 5′ end with [γ-^32^P]ATP and T4 polynucleotide kinase (New England Biolabs). Then, the reaction mixture was supplemented with 4 volumes of 1× hybridization buffer (150 mM KCl, 10 mM Tris pH 8.3, 1 mM EDTA) and subjected to gel filtration using Sephadex G-25 columns. Hybridization was carried in 20 μl containing 10 μg of total RNA, 3 μl of the labeled primer (5 × 10^5^ cpm) and 1× hybridization buffer. The mixture was denatured in 85 °C for 5 min. and incubated in 42 °C for 3 h. After addition of 0.1 volume of 3 M sodium acetate pH 5.2 and 2.5 volumes of absolute ethanol nucleic acids were precipitated overnight in −80 °C and then collected by centrifugation. Resulting pellet was washed in 80 % ethanol, air-dried and dissolved in 20 μl primer extension mixture containing: 1× buffer RT (Omniscript RT Kit, Qiagen), 10 mM DTT, 1 mM dNTPs, 1 μg of actinomycin D, 10 U of RNase inhibitor and 4 U of Omniscript reverse transcriptase (Qiagen). The reaction mixture was incubated at 42 °C for 2 h, treated with RNaseA and then extracted with phenol/chloroform. After extraction, the extension products were precipitated with ethanol and dissolved in 5 μl of stop solution from Sequenase DNA Sequencing Kit (USB). This kit was also used to produce a sequencing ladder for which the same primer and homologous DNA template were taken.

### Primers

All primers were purchased from specialized oligonucleotide manufacturers in deprotected and desalted form. Their sequences were as follows (5′ → 3′):

F, TAA GTG GCT CAC GAG GAA TG

G, AAG CGT GAC GAG AAT TCT CT

R, CTA CAT ATC ATA TCA T

U, CGC CAA AGC AAT TGT AGC AG

W, AGA GAA TTC TCG TCA CGC TT

ac-f, CTG TCT CTA TAT GCT AGT GG

ac-r, GAA TCT TCA TCA AGC CAT CT

Sequence of primers B, C, D and E is given in Szklarczyk et al. (2000).

### Production of anti-ATP9 antibodies and immunoblotting

Existing cDNA sequence data allowed design and custom-synthesis of a peptide, which corresponded to the following portion of carrot ATP9 protein: HSVARNPSLAKQLFGYA. Using glutaraldehyde as a crosslinker, this peptide was coupled to an ovalbumin carrier according to the procedure of Carter (1996). Unreacted glutaraldehyde was eliminated with addition of glycine to 10 mM. Resulting conjugate was dialyzed overnight at 4 °C against 4 L of PBS. Polyclonal antibodies were raised in a rabbit at antibody production unit of Polish—American Children’s Hospital (Jagiellonian University Medical College, Cracow, Poland). Appropriate antibody titer in the host animal’s serum was evidenced using ELISA (Peters and Baumgarten 1992).

Total hydrophobic proteins were extracted according to Michon et al. (1988) from 0.8 g of plant tissue powdered in liquid nitrogen. Ether-precipitated proteins were dissolved in 60 μl of the sample buffer (0.32 M Tris pH 6.8, 4 % glycerol, 8 % SDS, 0.1 M EDTA, 0.4 M DTT and 0.2 % bromophenol blue) and run in 14 % polyacrylamide gel using Tris-Tricine system of Schägger and von Jagow (1987). Subsequently, for 30 min., the proteins were semi-dry blotted onto nitrocellulose membranes using Trans-Blot SD unit (Bio-Rad) set at 10 V. After 2 h blocking in TBS-dissolved 3 % blocking reagent (Roche), the membranes were washed 3 times for 10 min. in TBS with 0.05 % Tween 20 and then incubated overnight in the anti-ATP9 serum (see above) diluted 100-fold in TBS containing 0.05 % Tween 20 and 1 % blocking reagent. After that, the washing steps were repeated and followed by 1.5 h incubation in anti-IgG Ab-alkaline phosphatase conjugate diluted 5,000-fold in TBS/0.05 % Tween 20/1 % blocking reagent. Further membrane processing included again triple 10 min. washing in TBS/0.05 % Tween 20, single 10 min. wash in TBS and equilibration in alkaline phosphatase buffer—3 times for 2 min. Color development was performed with the use of BCIP/NBT Liquid Substrate Plus (MP Biomedicals) and terminated by immersing the membrane in distilled water. The transfer buffer and alkaline phosphatase buffer were prepared according to Harlow and Lane (1988). Dry membranes were scanned in grayscale, and integrated optical density of the recorded signals was measured using ImageJ 1.33u (Wayne Rasband).

## Results

### Heterogeneity of genomic *atp9* sequences

**Fig. 1:**
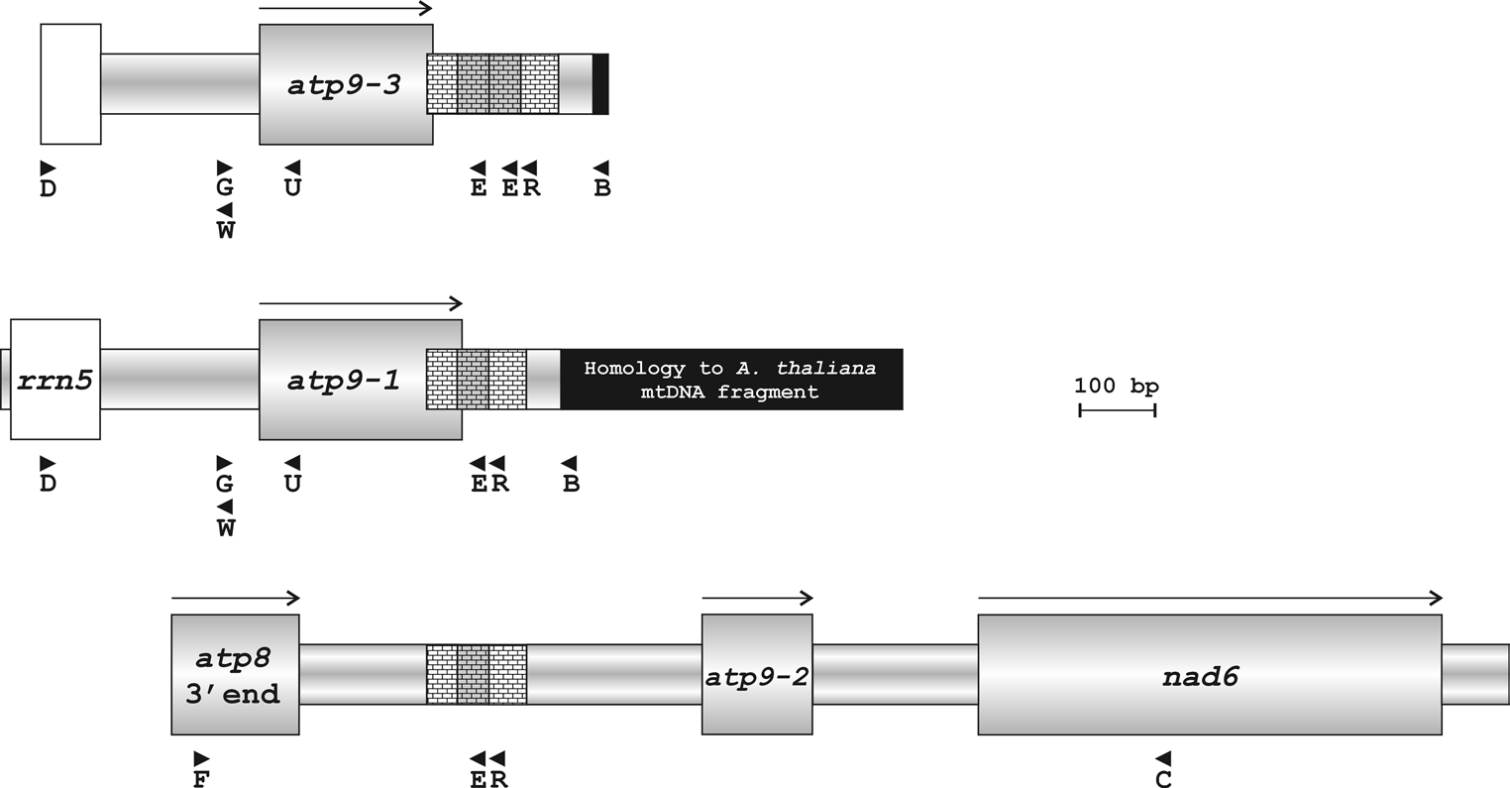
Sequence organization of carrot *atp9* loci. *Open boxes*, sequence of the *rrn5* gene; *shaded boxes*, open reading frames (ORFs) or their fragments; *black segments*, sequence of homology to the region of *Arabidopsis thaliana* mtDNA (nucleotides 16,342–16,816 of Y08501 record); *reticulate pattern*, sequence of the 130-/172-bp repeat; *shaded segments* *of* *reticulate pattern*, units of 42-bp duplication; *arrows*, ORF orientation;* arrowheads*, location of primers

**Fig. 2:**
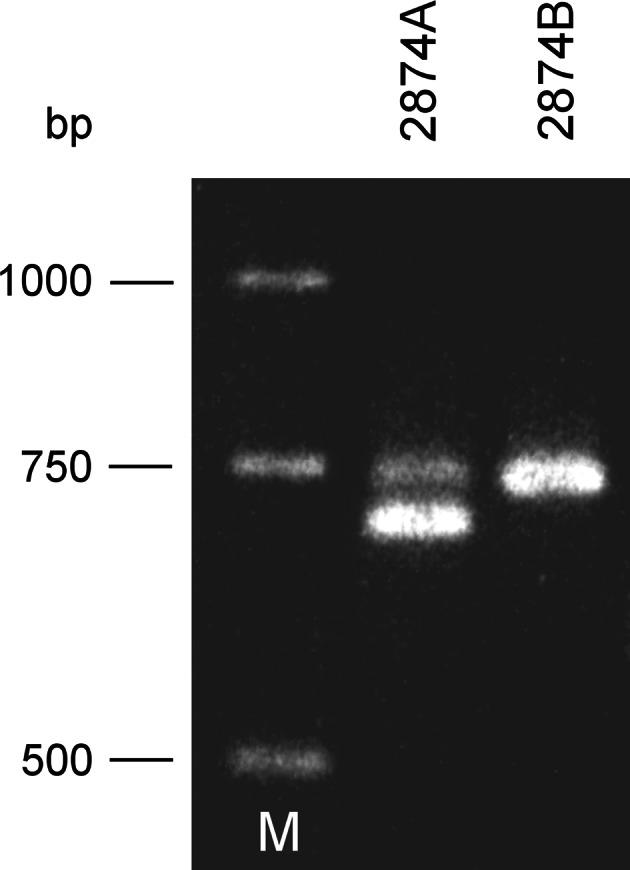
PCR-amplified *atp9* sequences from lines 2874A and 2874B with primers B and D. M, DNA size standard

Due to duplication of the 42-bp stretch at the 3′ end of *atp9*-*3* (Szklarczyk et al. 2000; Fig. 1), this gene can be easily differentiated from *atp9*-*1* using PCR. Therefore, a PCR experiment was performed using primers D and B—anchored, respectively, upstream and downstream of either *atp9* open reading frame (ORF). Total genomic DNA from lines 2874A (petaloid CMS) and 2874B (fertile) was used as a template in these reactions. According to expectations, line 2874B yielded a single product of 0.75 kb (Fig. 2). Consequently, a smaller product was observed for line 2874A—its size was about 0.71 kb. However, the latter product was accompanied by another DNA fragment—co-migrating with the one of 0.75 kb obtained for the male-fertile line. Both bands observed in petaloid carrots were different in their intensity indicating much weaker amplification of the longer DNA fragment.

The above results indicate that both versions of the *atp9* sequence are present in petaloid carrots. The two products obtained for line 2874A with primers D and B were cloned and sequenced. The results confirm the presence of both *atp9* versions in the petaloid material (electronic supplementary material, Fig. S1). In order to determine relative abundance of both *atp9* sequences, another PCR experiment was performed. Total genomic template from 3 CMS inbreds and their maintainers was used with primers G and E anchored in closer vicinity to either *atp9* ORF (Fig. 1). Resulting amplification products were cloned in a plasmid vector, and sequence of 9–13 recombinant clones was determined for each analyzed line. Distinction between *atp9*-*1* and *atp9*-*3* was made on the basis of the 42-bp duplication at the 3′ end and/or on the basis of two single nucleotide polymorphisms (SNPs) located between the anchor sites of primers G and E (Fig. S1). In accordance with the previous experiment (see above), all clones derived from the male-fertile B lines contained the sequence of *atp9*-*3* (Table 1). Conversely, among clones produced for the male-sterile 2163A line, only the sequence of *atp9*-*1* was found. Likewise, the *atp9*-*1* gene was present in the majority of the clones from A88A and 2874A. However, for each of these lines, one clone representing *atp9*-*3* sequence was also noted. These results confirmed the PCR pattern produced with primers B and D (Fig. 2).

**Table 1:** Number of genomic *atp9*-*1* and *atp9*-*3* molecules in the sampled clones from male-sterile (A) and male-fertile (B) carrot lines

Sequence → Line ↓	*atp9*-*1*	*atp9*-*3*
A88A	11	1
A88B	0	13
2163A	10	0
2163B	0	10
2874A	11	1
2874B	0	9

### Recombination involving the *atp9* loci

Comparison of mtDNA regions, which are adjacent to carrot *atp9* genes, revealed the presence of a repeated sequence stretch at the 3′ end of both *atp9*-*1* and *atp9*-*3* as well as upstream of the *atp9*-*2* pseudogene (Fig. 1). The copies located in the *atp9*-*1* and *atp9*-*2* loci are 130-bp long, while in case of *atp9*-*3*, due to internal 42-bp duplication, the repeat reaches a length of 172 bp. Recombination activity of the 130-/172-bp repeat could produce alternative sequence arrangements around this DNA segment. In order to verify this hypothesis, two PCRs were performed with primers oriented toward the repeat and genomic DNA template from lines 2874A and 2874B.

**Fig. 3:**
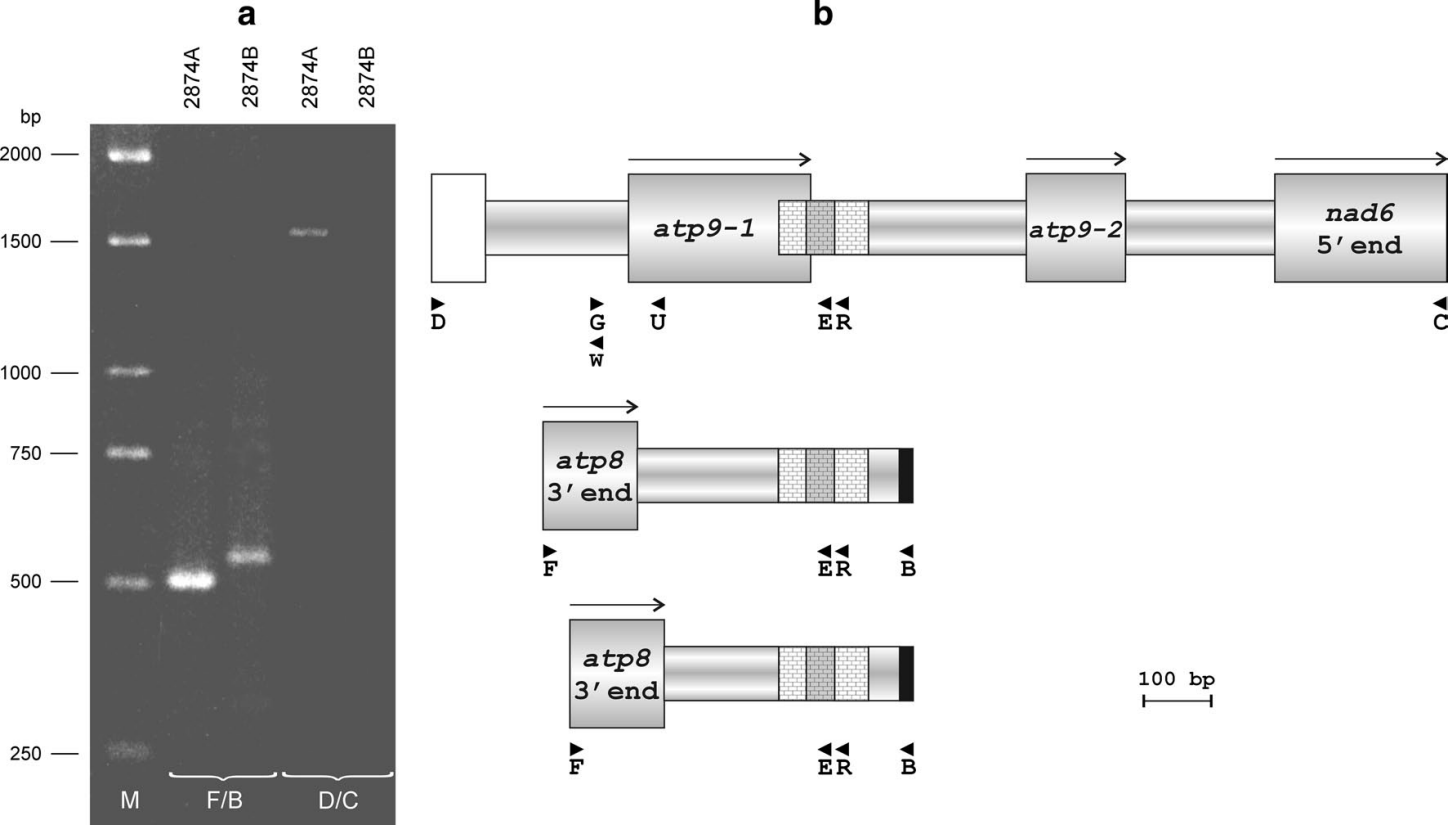
**a** PCR-based detection of recombinant sequence arrangements around the 130-/172-bp repeat identified in the vicinity of carrot *atp9* genes. Source of template and primer combinations are indicated above the gel lanes and at the bottom of the picture, respectively. M, DNA size standard. **b** Schematic sequence organization of the PCR products.* Open box*, fragment of the *rrn5* gene; *shaded boxes*, open reading frames (ORFs) or their fragments;* black segments*, terminal part of the sequence with homology to the region of *Arabidopsis thaliana* mtDNA (see Fig. 1); *reticulate pattern*, sequence of the 130-/172-bp repeat; *shaded segments* *of* *reticulate pattern*, units of 42-bp duplication; *arrows*, ORF orientation;* arrowheads*, location of primers

**Fig. 4:**
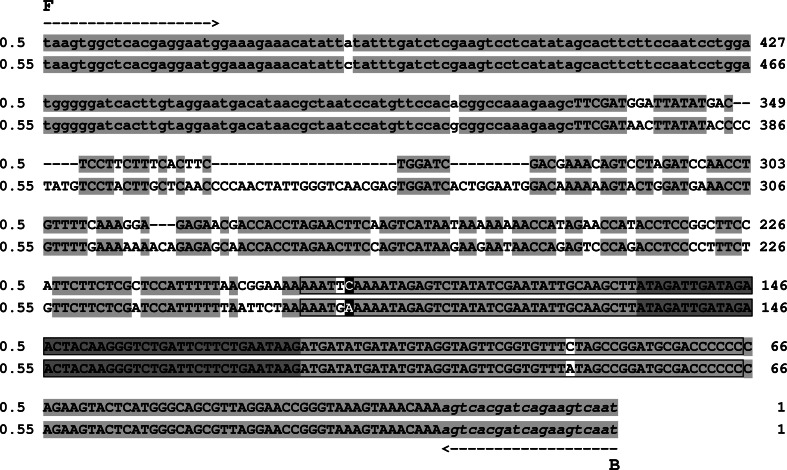
Aligned sequences of the 0.5 and 0.55 kb PCR fragments produced with the use of primers F and B for lines 2874A and 2874B, respectively. *Light shading*, sequence identity; *dark shading*, the unit of 42-bp duplication;* black shading*, nucleotides corresponding to these which differentiate *atp9*-*1* and *atp9*-*3* ORFs;* boxed letters*, sequence of the 130-bp repeat; *lowercase letters*, sequence of homology to *atp8* genes; *lowercase italics*, terminal part of the sequence with homology to the region of *Arabidopsis thaliana* mtDNA (see Fig. 1); *arrows*, primer binding sites

The first reaction was carried with primers F and B, the former anchored within *atp8* sequence located upstream of *atp9*-*2* and the latter within the sequence of homology with a fragment from the *Arabidopsis thaliana* mtDNA. Using this primer combination, PCR products were obtained for both lines (Fig. 3a). Their sizes were 0.5 and 0.55 kb for the male-sterile (2874A) and male-fertile (2874B) line, respectively. Both products were sequenced using primers F and B. The sequence features of both DNA fragments are described as follows using nucleotide numbering with reference to the terminus produced with primer B. Upon sequence alignment (Figs. 3b, 4), the products revealed a stretch of near-identity over a distance of 190 bp from the terminus produced with primer B—in this region, they differ only in position 86. This 190-bp sequence is also present at the 3′ end of *atp9*-*1* and *atp9*-*3*. The polymorphic position 86 mentioned above corresponds to the nucleotide, which also differentiates 3′ flanking regions of these genes. In case of *atp9*-*1* and the 0.5 kb product, this position is occupied by a cytosine, while *atp9*-*3* and the product of 0.55 kb exhibit the presence of an adenine. Moreover, cytosine in the position 165 of both products matches either a cytosine within 3′ flanking region of *atp9*-*1* or a thymine in the analogous fragment of *atp9*-*3*. When proceeding along the sequence from the sequence of primer B, close similarity of both products ends in the position 191. This corresponds to nucleotide 229 of the *atp9*-*1* and *atp9*-*3* open reading frames (ORF), which determines either presence or absence of the 13 amino acid C-terminal extension (Szklarczyk et al. 2000). Similar to the *atp9*-*1* ORF, the product of 0.5 kb contains here a cytosine, whereas the *atp9*-*3* ORF and the 0.55 kb product contain a thymine and an adenine, respectively.

High sequence similarity of both products is also observed over 146 bp extending from the F primer terminus, where only two single nucleotide substitutions are present. This region shows homology to plant mitochondrial *atp8* genes. Both products differ mostly in the central part of their sequence, which is also responsible for their size difference. By summarizing the features of the 0.5 kb product, it becomes apparent that it is fully colinear with the respective sequences in the vicinity of *atp9*-*1* and *atp9*-*2*.

The second reaction was performed with the use of primers D and C. The former was anchored within the *rrn5* gene located upstream of both *atp9*-*1* and *atp9*-*3*, the latter—within the *nad6* ORF, which was found downstream of the *atp9*-*2* pseudogene (Fig. 1). The resulting PCR product was about 1.5 kb long and appeared only for 2874A (Fig. 3a). This product was sequenced using primer walk strategy starting with the primers used for its amplification. Sequence examination revealed full colinearity of this product with the respective segments of the *atp9*-*1* and *atp9*-*2* loci found in the library clones. Therefore, the product contained the fragment of *rrn5* gene, the *atp9*-*1* ORF with adjoining 130 bp of the repeat unit and the *atp9*-*2* pseudogene followed by the 5′ segment of *nad6* (Fig. 3b).

The genuine character of the above-described amplicons was confirmed with the use of long PCR and Southern hybridization (electronic supplementary material, Figs. S2, S3).

### Heterogeneity of *atp9* mRNAs, their editing pattern and 5′ termini

In the next series of experiments, the relative abundance of both carrot *atp9* sequences was examined at the RNA level. For this purpose, cDNA was produced for the set of inbreds previously used to analyze genomic *atp9* heterogeneity (see above). Subsequently, the cDNA preparations were used as a template for PCR with primers G and E. As before, resulting amplification products were cloned and sequences of several randomly picked clones were determined. Upon cDNA sequence examination, it was noted that the CMS A accessions contained only transcripts of the mutated gene—*atp9*-*1* (Table 2). In one of the male-fertile inbreds—2874B—exclusively non-mutated *atp9*-*3* cDNAs were found. This sequence was prominent also among the cDNA clones from two other male-fertile lines—A88B and 2163B. However, the latter cDNA populations also contained a certain small fraction (altogether 24 % of the examined molecules) of the mutated *atp9*-*1* sequences.

**Table 2:** Number and editing pattern of cDNA molecules representing *atp9*-*1* and *atp9*-*3* sequences in the sampled clones from male-sterile (A) and male-fertile (B) carrot lines

No. of molecules	Sequence	Edited nucleotide (position in relation to A of AUG start)				
		−4	20	205	215	223
Line A88A						
8	*atp9*-*1*		+	+	+	+
4	*atp9*-*1*	+	+	+	+	+
Line A88B						
5	*atp9*-*3*		+	+	+	+
1	*atp9*-*3*		+			
4	*atp9*-*1*		+	+	+	+
Line 2163A						
12	*atp9*-*1*		+	+	+	+
Line 2163B						
6	*atp9*-*3*		+	+	+	+
1	*atp9*-*3*	+	+	+	+	+
1	*atp9*-*3*		+		+	
1	*atp9*-*3*		+	+	+	
1	*atp9*-*3*		+		+	+
1	*atp9*-*1*		+	+	+	+
Line 2874A						
11	*atp9*-*1*		+	+	+	+
Line 2874B						
11	*atp9*-*3*		+	+	+	+
1	*atp9*-*3*		+		+	+

Alignment of the genomic and cDNA sequences revealed 4 C to U editing sites within the open reading frame of the *atp9* mRNAs (Table 2). Interestingly, all *atp9*-*1* molecules exhibited complete editing in contrast to those of the *atp9*-*3* type, among which 18 % was edited partially. Consequently, partial editing was observed only in the male-fertile accessions. Editing in positions 20 and 215 leads to conversion of serine triplets into leucine and phenylalanine codons, respectively (Table 3). The change in position 205 is silent. Conversion in position 223 turns a CAA glutamine triplet into the UAA stop codon. Therefore, assuming complete editing, cDNA-encoded ORFs appear to be shorter than their genomic counterparts, which makes them identical for both versions of the *atp9* gene. Another editing site was identified 4 nucleotides upstream of the *atp9* ORF. This event was noted for 5 out of the 68 analyzed cDNA clones.

**Table 3:** Influence of RNA editing on coding features of carrot *atp9* ORFs

Nucleotide position	Codon position	Codon	Codon meaning change
−4	–	–	–
20	7	UCA	Ser → Leu
205	69	CUG	–
215	72	UCU	Ser → Phe
223	75	CAA	Gln → stop

**Fig. 5:**
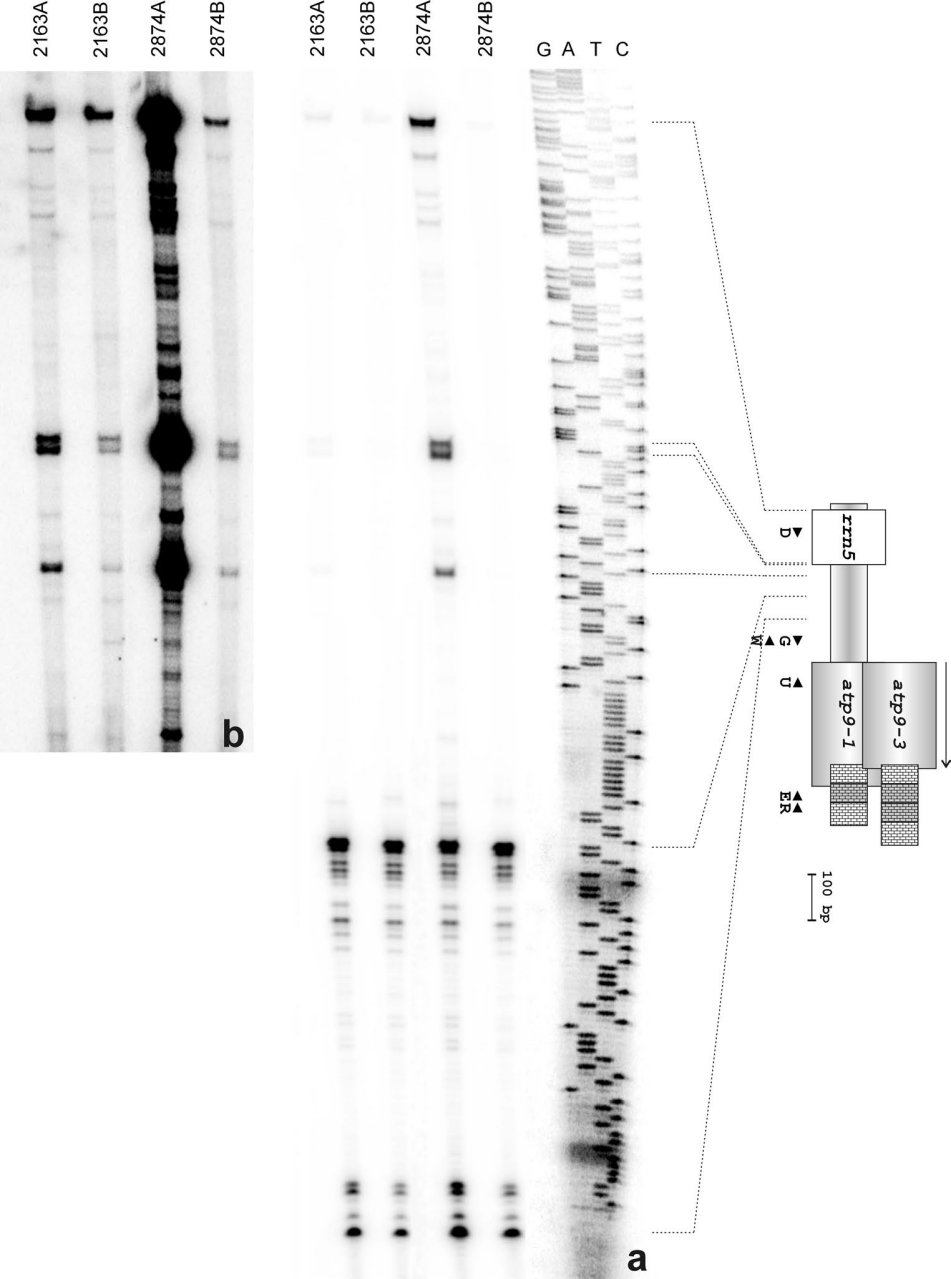
**a** Mapped 5′ termini of carrot *atp9* transcripts. Primer extension analysis was carried with the use of primer W and RNA preparations from lines 2163A, 2163B, 2874A and 2874B. The sequence ladder covers a part of the *atp9*-*rrn5* intergenic spacer as well as the *rrn5* gene itself. Schematic representation of the *atp9* loci is shown on the right (for description see Fig. 1). **b** Enhanced exposure of the upper part of the gel

To determine whether sequence heterogeneity is accompanied by diversity in transcript initiation/processing sites, a series of primer extension reactions was performed. In these experiments, two primers were used: U—anchored within 5′ part of the *atp9* coding sequence (Fig. 1) and W—positioned within the spacer region between genes *atp9* and *rrn5*. The sequence ladders produced with these primers covered the *atp9* upstream sequences reaching behind the 5′ end of the *rrn5* coding region. Both oligos were used to prime cDNA synthesis from mRNA of plants representing lines 2163A, 2163B, 2874A and 2874B. The mapped 5′ termini fell into two categories (Fig. 5a): (i) those common to all studied mRNA samples—represented by 2 major and several less pronounced signals positioning in the central part of the *atp9*/*rrn5* spacer; (ii) those specific for 2874A—they mapped further upstream, one in the vicinity and another 3 at the borders of the *rrn5* sequence. However, upon enhanced exposure, bands corresponding to the second category of termini became visible not only in the lane of 2874A but also for 2163A and to a lesser extent, for the male-fertile B lines (Fig. 5b).

### Quantitation of the *atp9* sequences and accumulation of their protein product

**Fig. 6:**
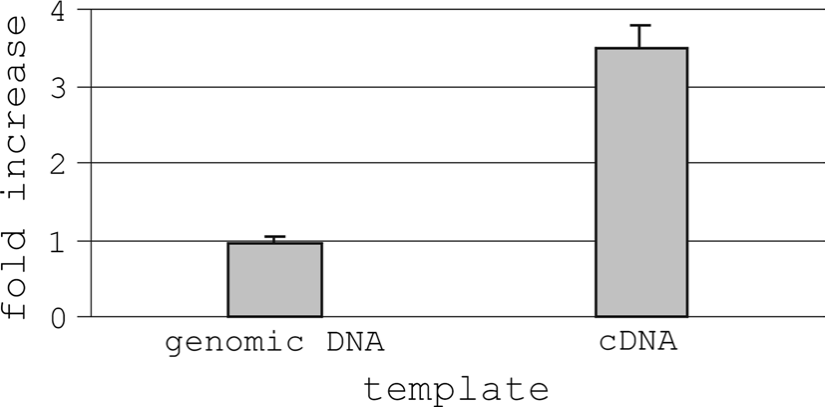
Relative quantitation of *atp9* sequences based on real-time PCR. Bars represent a fold increase in amplicon content observed in line 2163A versus line 2163B (calibrator). The results were normalized using C_t_ values obtained for actin gene as an endogenous control

Relative quantitation of the *atp9* sequences was performed on both genomic and cDNA level with the use of real-time PCR. The reactions were driven with primers G and U, which accessed either of the analyzed *atp9* genes. The relative increase of the target copy number was calculated for the line 2163A versus 2163B. It turned out that copy number of the genomic *atp9* sequence is comparable in the S_p_- and N-cytoplasmic plants (Fig. 6). However, these forms differed substantially with respect to amount of the *atp9* transcript—its level appeared to be more than three times higher in case of the CMS line. The respective ΔΔC_t_ values were 0.05 (±0.09) and −1.8 (±0.13) for genomic and cDNA template, respectively.

**Fig. 7:**
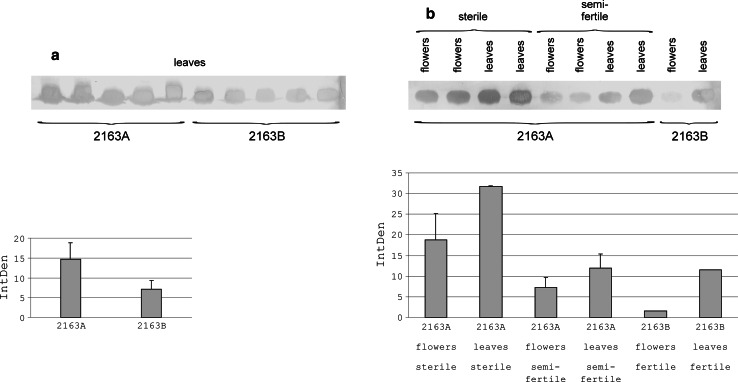
Western blot analysis of carrot hydrophobic proteins probed with anti-carrot *ATP9* serum. Source plants originated from lines 2163A and 2163B. **a** Proteins extracted from leaves of plants at the vegetative stage, **b** proteins extracted from flowers and leaves of flowering plants. Densitometric measurement of the resulted signals is shown in graphs below

Accumulation of the ATP9 protein was also compared for lines 2163A and 2163B. In the first immunoblotting experiment, the anti-ATP9 serum was used to probe preparations of hydrophobic proteins from leaves of plants at the vegetative stage (Fig. 7a). The signals obtained for the S_p_-cytoplasmic carrots were approximately twofold stronger than those for plants carrying the N-cytoplasm. The second experiment was performed using protein preparations from flowering plants. In this case, phenotype assessment within 2163A allowed selection of occasional semi-fertile plants. Therefore, it was possible to compare male-sterile and semi-fertile S_p_-cytoplasmic plants in the context N-cytoplasmic control (Fig. 7b). In accordance with the first immunoblotting experiment, over twofold reduction in signal strength was observed in the semi-fertile versus male-sterile plants. The difference was observed for both leaves and flowers. These data corresponded to the reduced signal intensity noted for the N-cytoplasmic controls.

## Discussion

In 2000, Szklarczyk et al. reported that CMS carrots carrying S_p_-cytoplasm contained a mutated version of the *atp9* gene (*atp9*-*1*) instead of its normal counterpart (*atp9*-*3*) present in male-fertile N-cytoplasmic accessions. Our new data show that, instead of being cytoplasm-specific, these genes function in the heteroplasmic condition and that differences in their distribution are simply quantitative. Sequences representing the *atp9*-*1* version predominate in petaloid carrots (S_p_-cytoplasm). In these forms, the *atp9*-*3* gene comprises less that 10 % of the total functional *atp9* complement. In contrast, in N-cytoplasmic plants the *atp9*-*3* version dominates over *atp9*-*1*. Presence of the latter was undetectable on genomic level but the respective molecules were successfully amplified using cDNA template. Described cases of plant mitochondrial heteroplasmy usually refer to the coexistence of normal and rearranged genome variants (Janska et al. 1998; Newton et al. 2004). Due to extremely low mutation rates in plant mitochondrial genes, little attention has been directed toward intraspecies sequence heterogeneity (Städler and Delph 2002). Therefore, our knowledge about heteroplasmic status of minor-scale sequence variants is only emerging (Hattori et al. 2002) with this case in carrot among the first revealed.

The carrot *atp9* genes show that extreme care must be taken in considering the homoplasmic status of a plant mitochondrial sequence. This is best illustrated by the fact that the *atp9*-*1* variant was not detected on genomic level in the normal cytoplasm. Without examination of the RNA pool and specific features of the *atp9*-*1* transcript (discussed below), the N-cytoplasm would be regarded as homoplasmic with the sole presence of *atp9*-*3*. Sequence heterogeneity is especially easy to miss when direct sequencing of the respective PCR products is applied. Some evidence was also presented in literature to indicate transient character of heteroplasmic status (Grun 1976). Taking into account the distinct origin of the S_p_- and N-cytoplasm and presence of either *atp9* version in both, it appears that the heteroplasmic status in plants can be quite persistent.

With the above considerations, it seems that differentiation of the S_p_-cytoplasm, most likely from the N-like progenitor, was accompanied by a stoichiometric shift in abundance of the *atp9* sequence variants. Upon this shift, a sublimon-type *atp9*-*1* version eventually became the prevailing functional *atp9* sequence in the genome. Lack of the untypical C-terminal extension in the *atp9*-*3* open reading frame (ORF) favors assumption that this represents the original sequence variant. Moreover, the organization of the *atp9* loci was influenced by the recombination activity of the 130-/172-bp repeat. Such dispersed sequence repeats can result from non-functional mRNA fragments, which after reverse transcription, are integrated into new genomic localizations (André et al. 1992). Presence of the repeat unit at the 3′ end of both *atp9* ORFs seems to confirm this scenario and secondary character of the repeat copy located near the *atp9*-*2* pseudogene. Recombination between these two repeat copies led to formation of the sequence arrangements represented by the PCR fragments produced with the primer pairs F/B and D/C. Since the former combination yielded different products for the S_p_- and N-cytoplasmic accession, either we can infer that this reflects an ancient character with subsequent accumulation of the sequence differences in both variants or alternatively, their independent formation in the S_p_ and N-cytoplasm. These considerations can include several possibilities explaining how the *atp9*-*1* version was actually formed, the more so because effects of the repeat-induced and intragenic recombination (already postulated for plant mtDNA, Städler and Delph 2002) here may act in superposition.

Reporting their physical mapping work, Robison and Wolyn (2002) found that only assuming recombination across short repeats, it would be possible to represent the entire carrot mitochondrial genome in the form of circular molecules. They pointed at the 60-bp inverted repeat found upstream of *atp8* (*orfB*) and downstream of the *atp9* gene as a potential candidate for such recombination, but they did not detect the respective genomic environments. Here, we provide this evidence for the 130-/172-bp repeat originally found downstream of both *atp8* (*orfB*) and *atp9* (*atp9*-*1*)—so in the similar genomic context.

Extensive heteroplasmy reported for the *nad3*-*orf156* locus in wheat was not accompanied by the respective heterogeneity of the *orf156* transcripts (Hattori et al. 2002). Contrary to that observation, it appears that carrot heteroplasmy of the *atp9* sequence is transmitted to the mRNA level. Presence of both sequence variants was revealed within the transcript pool in two of the three studied N-cytoplasmic inbreds. This result was surprising considering that the *atp9*-*1* version was not traceable in the normal cytoplasm at the genomic level. On the other hand, for lines carrying the S_p_-cytoplasm, in which heteroplasmic status was demonstrated clearly, exclusive detection of the *atp9*-*1* mRNAs was obtained. However, all these facts are easily explainable if one assumes that the *atp9*-*1* transcripts exhibit either higher rate of synthesis, increased stability or a combination of both these features. Such an assumption is strongly supported by the results of real-time PCR showing that overall accumulation of the *atp9* mRNA is much higher in the S_p_-cytoplasm. Those collected data indicate that the *atp9*-*1* mRNAs are robust enough to manifest the presence of their otherwise undetectable genomic template in the N-cytoplasm and to displace the *atp9*-*3* transcripts despite detectable amounts of their template in the S_p_-cytoplasm.

The unique character of both transcripts is emphasized by the fact that partially edited copies were found exclusively among the pool of the *atp9*-*3* mRNAs. All examined *atp9*-*1* transcripts exhibited complete ORF editing, even if they originated from N-cytoplasmic plants. Efficiency of editing varied as a function of plant development, growth conditions and nuclear background (for ref. see Gagliardi and Gualberto 2004). In addition to those data, in the carrot system examined here, mRNA behavior was largely attributable to the transcript’s own characteristics. Since partial editing was only noted for positions which are located in the vicinity (within 24 upstream nucleotides) of the nucleotide 229 which differentiates both *atp9* ORFs, it is possible that this sequence difference influences efficiency of editing. This interpretation complements observations that editing machinery recognizes stretches of 20–25 nucleotides mostly upstream of the editing site when defining the nucleotide to be edited (Takenaka et al. 2013). It is also possible that observed differences in editing efficiency are derivatives of differential processing of the *atp9*-*1* and *atp9*-*3* mRNAs, similar to the situation shown for the *B*-*atp6* transcripts in rice (Iwabuchi et al. 1993). Existence of processing differences was shown with the use of primer extension analysis that demonstrated that signals for the termini mapping more distantly from the *atp9* ORF were much stronger in the CMS lines in which only the *atp9*-*1* transcripts were detected. Indication that all mapped 5′ extremities represent rather processing than initiation sites comes from the observation that the three most upstream termini border the sequence of the adjoining *rrn5* gene. This suggests that the respective processing events liberate mature 5S rRNA and that carrot *atp9* genes do not have their own promoter but rather form one transcription unit with the *rrn5* and very likely—also the *rrn18* sequence. Co-transcription of these two rRNA genes has also been observed in other studied plant species (Binder et al. 1996). As was also seen from the primer extension data, and contrary to the previous RT-PCR-based report (Szklarczyk et al. 2000), co-transcription of *atp9* and its upstream sequences is not restricted to the S_p_-cytoplasm, since the respective signals were also recorded for the N-cytoplasmic lines upon prolonged gel exposure. This may be either enhanced production or increased stability of the (*rrn18*-) *rrn5*-*atp9* co-transcripts, which accounts for their greater abundance in petaloid carrots. The relevance of mRNA stability is supported by the fact that Sp- and N-cytoplasmic plants differ also with respect to 3′ transcript extremities. The 3′ termini were probed using RT-PCRs with a set of reverse primers covering the region downstream of the *atp9* ORFs (data not shown). In plants carrying the N-cytoplasm, the most far-reaching primer, for which amplification was observed, was anchored within the distal part of the 42-bp repeat unit (Fig. 1). In the S_p_-cytoplasmic plants, the RT-PCR products were generated with reverse primers located as far as in the central part of the region with homology to *Arabidopsis thaliana* mtDNA (approximately 0.3 kb from the 42-bp repeat). For the S_p_-cytoplasm, the broader span of transcript extremities agrees with detection of the specific high molecular *atp9* mRNAs by RNA blot hybridization (Szklarczyk 1997; Szklarczyk et al. 2000). These specific transcripts were always accompanied by smaller mRNA species which occurred also in the N-cytoplasm suggesting that the low-size transcripts resulted from processing of the large precursors which exhibited increased stability in case of the petaloid plants. If there is a temporal correlation between RNA editing and processing (Gagliardi and Gualberto 2004), increased stability may also explain the lack of partially edited molecules among the *atp9*-*1* transcript pool. Despite the sequence variation between the *atp9*-*1* and *atp9*-*3* loci, its significance for altered transcript stability remains speculative. RNA folding predictions performed for the 3′ flanking regions of both carrot *atp9* variants indicate that anticipated secondary structures differ mostly due to the status of the 42-bp repeat (data not shown). Since stem-loop structures formed at 3′ ends of plant mitochondrial mRNAs correlate with transcript stability (Williams et al. 2000 and references therein), it is likely that this sequence polymorphism is responsible for the observed differences between transcripts of *atp9*-*1* and *atp9*-*3*.

Four of the 5 editing sites identified are located within the *atp9* ORF. Editing at nucleotide positions 20, 205 and 223 was previously found in other plant species (Dell’Orto et al. 1993; Laser et al. 1995), while the event at position 215, to our knowledge, has not been demonstrated before. A number of additional edit sites are known for the *atp9* transcripts in other plants—they include positions: 50, 81, 82, 90, 92, 134, 182, 191 and 212 (see above ref.). In the carrot genes, all of these sites are pre-edited, thereby already occupied by a thymine, and therefore, they do not require C to U conversion at the RNA level. Overall, the editing events make the deduced sequence of the ATP9 protein like analogous sequences from other plant species (data not shown). The compensatory effect of editing is also manifested by elimination of the sequence difference between the ORFs of *atp9*-*1* and *atp9*-*3*. Therefore, this observation also prompted us to test if the *atp9* sequences display any quantitative differences in expression.

Our present data show that, while overall copy numbers of the functional *atp9* genes are comparable in the S_p_ and N-cytoplasms, the former exhibits a pronounced over-expression at the RNA and particularly at the protein level. Increased levels of mitochondrial mRNAs have been reported for alloplasmic lines of *Brassica rapa* carrying the cytoplasm of *Diplotaxis muralis* (Yamasaki et al. 2004). The authors postulate that this enhanced expression results from novel nucleo-mitochondrial interactions. Since the petaloid CMS in carrots is also of alloplasmic origin, it is possible that similar effects contribute to the *atp9* over-expression reported here. At this point of our knowledge, it is difficult to specify whether the *atp9* over-expression and the CMS trait are causally related or that they simply co-occur. Since all CMS determinants characterized to date represent novel, aberrant mitochondrial sequences (Schnable and Wise 1998; Fujii and Toriyama 2008), this case of “quantitatively” conditioned CMS would be exceptional. According to the scenario we describe above, the status of enhanced ATP9 accumulation in the S_p_-cytoplasm is achieved most likely through the enhanced level of the *atp9*-*1* sequence and its expressional dominance over *atp9*-*3*. An assumption that expression of CMS in carrots relies on the dynamic mutual proportion between mutated and normal sequence factors could easily explain both cases of fertility reversion (Chahal et al. 1998) and induction of petaloidy through in vitro culture (Wright et al. 1996). In favor of a causal relationship between ATP9 over-expression and CMS is the reduced ATP9 accumulation, which was observed in the semi-fertile S_p_-cytoplasmic plants. So far such evidence has not been reported for another carrot candidate CMS factor, the altered *atp8* gene (Nakajima et al. 2001). However, we cannot exclude the possibility that the respective restorer gene might influence expression of loci, which are not related to CMS.

## Electronic supplementary material

Below is the link to the electronic supplementary material.

Supplementary material 1 (DOCX 14 kb)

Supplementary material 2 (DOCX 177 kb)

Supplementary material 3 (DOCX 194 kb)

## Abbreviations

*AP1*: *APETALA1*
*AP2*: *APETALA2*
*AP3*: *APETALA3*
ATP9: ATP synthase subunit 9
CMS: Cytoplasmic male sterility
C_t_: Threshold cycle
IntDen: Integrated optical density
mtDNA: Mitochondrial DNA
*PI*: *PISTILLATA*
ORF: Open reading frame
*SEP3*: *SEPALLATA3*
