# Supplementary material for: Mitochondrial atp9 genes from petaloid male-sterile and male-fertile carrots differ in their status of heteroplasmy, recombination involvement, post-transcriptional processing as well as accumulation of RNA and protein product
Source: Theor Appl Genet. 2014 Jun 10;127(8):1689–701. doi: 10.1007/s00122-014-2331-x (PMC4110418; doi:10.1007/s00122-014-2331-x)
Supplement: Supplementary file 1 — Supplementary material 1 (DOCX 14 kb) [file 122_2014_2331_MOESM1_ESM.docx]

**> 0.75 kb product (*atp9-3*)**

**cattccgacctcgatatgtggaatcgtcttgcgccatatgtactgagattgttcgggagacatggtccaagcccggtgaagaaatgaagccaacattgactgactttcatggttaagattcgacaaaaaagaaagaaaaaggattaagttagtgttaagtgagtgaaagatagctttatgacgagaggaggggaagagtctatcaaattgtgactctaaaacaaataaaagaaaaaggcgtgacgagaattctctaattagacaaagaattttattattagatacatacattatgttagaaggtgcaaaatcaataggtgccggagctgctacaattgctttggcgggagctgctattggaattggaaacgttttcagttctttgattcattctgtagcacgcaatccatcattggcaaaacaattatttggttatgccattttaggctttgcgctgacagaggctattgcattgtttgctctaatgatggcttttctgatcttatctgtattccaaatttaaaatagagtctatatcgaatattgtaagcttATAGATTGATAGAACTACAAGGGTCTGATTCTTCTGAATAAGATAGATTGATAGAACTACAAGGGTCTGATTCTTCTGAATAAGatgatatgatatgtaggtagttcggtgtttatagccggatgcgacccccccagaagtactcatgggcagcgttaggaaccgggtaaagtaaacaaaagtcacgatcagaagtcaat**

**> 0.71 kb product (*atp9-1*)**

**cattccgacctcgatatgtggaatcgtcttgcgccatatgtactgagattgttcgggagacatggtccaagcccggtgaagaaatgaagccaacattgactgactttcatggttaagattcgacaaaaaagaaagaaaaaagattaagttagtgttaagtgagtgaaagatagctttatgacgagaggaggggaagagtctatcaaattgtgactctaaaacaaataaaagaaaaaagcgtgacgagaattctctaattagacaaagaattttattattagatacatacattatgttagaaggtgcaaaatcaataggtgccggagctgctacaattgctttggcgggagctgctattggaattggaaacgttttcagttctttgattcattctgtagcacgcaatccatcattggcaaaacaattatttggttatgccattttaggctttgcgctgacagaggctattgcattgtttgctctaatgatggcttttctgatcttatctgtattccaaattcaaaatagagtctatatcgaatattgcaagcttATAGATTGATAGAACTACAAGGGTCTGATTCTTCTGAATAAGatgatatgatatgtaggtagttcggtgtttctagccggatgcgacccccccagaagtactcatgggcagcgttaggaaccgggtaaagtaaacaaaagtcacgatcagaagtcaat**

**xxxxx primer D anchor**

**xxxxx primer B anchor**

**xxxxx primer G anchor**

**xxxxx primer E anchor**

**xxxxx *atp9* ORF**

**xxxxx SNPs used to differentiate between *atp9-1* and *atp9-3***

**in the products amplified with primers G and E**

**XXXXX 42-bp repeat unit (second copy in *atp9-3* underlined)**

Fig. S1. Sequence features of the amplification products obtained from line 2874A with primers D and B.
