# Supplementary material for: Mitochondrial atp9 genes from petaloid male-sterile and male-fertile carrots differ in their status of heteroplasmy, recombination involvement, post-transcriptional processing as well as accumulation of RNA and protein product
Source: Theor Appl Genet. 2014 Jun 10;127(8):1689–701. doi: 10.1007/s00122-014-2331-x (PMC4110418; doi:10.1007/s00122-014-2331-x)
Supplement: Supplementary file 2 — Supplementary material 2 (DOCX 177 kb) [file 122_2014_2331_MOESM2_ESM.docx]

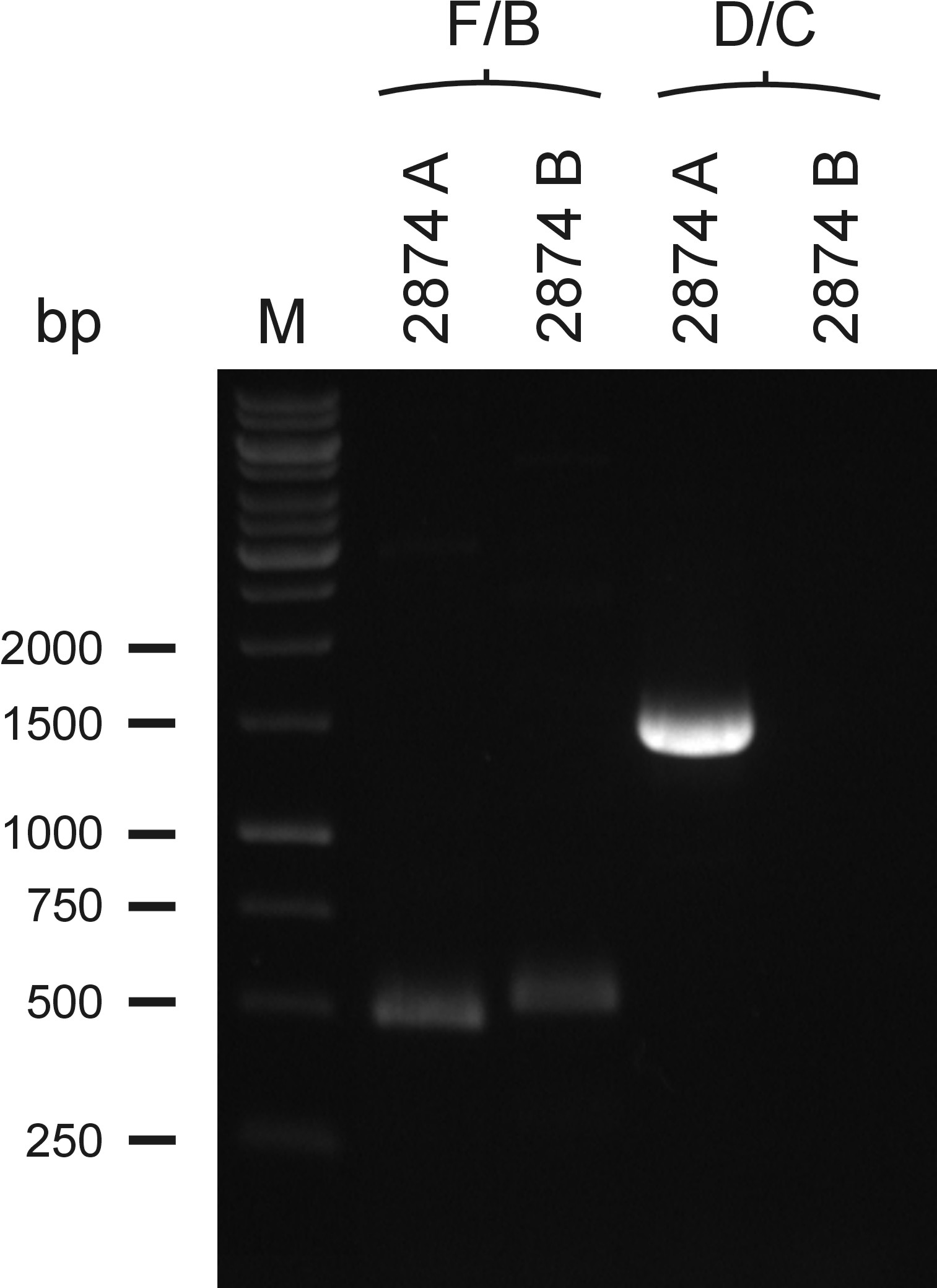


Fig. S2. Long PCR verification of the amplicons generated from lines 2874A and 2874B with the use of primer combinations F/B and D/C.
