# Supplementary material for: Mitochondrial atp9 genes from petaloid male-sterile and male-fertile carrots differ in their status of heteroplasmy, recombination involvement, post-transcriptional processing as well as accumulation of RNA and protein product
Source: Theor Appl Genet. 2014 Jun 10;127(8):1689–701. doi: 10.1007/s00122-014-2331-x (PMC4110418; doi:10.1007/s00122-014-2331-x)
Supplement: Supplementary file 3 — Supplementary material 3 (DOCX 194 kb) [file 122_2014_2331_MOESM3_ESM.docx]

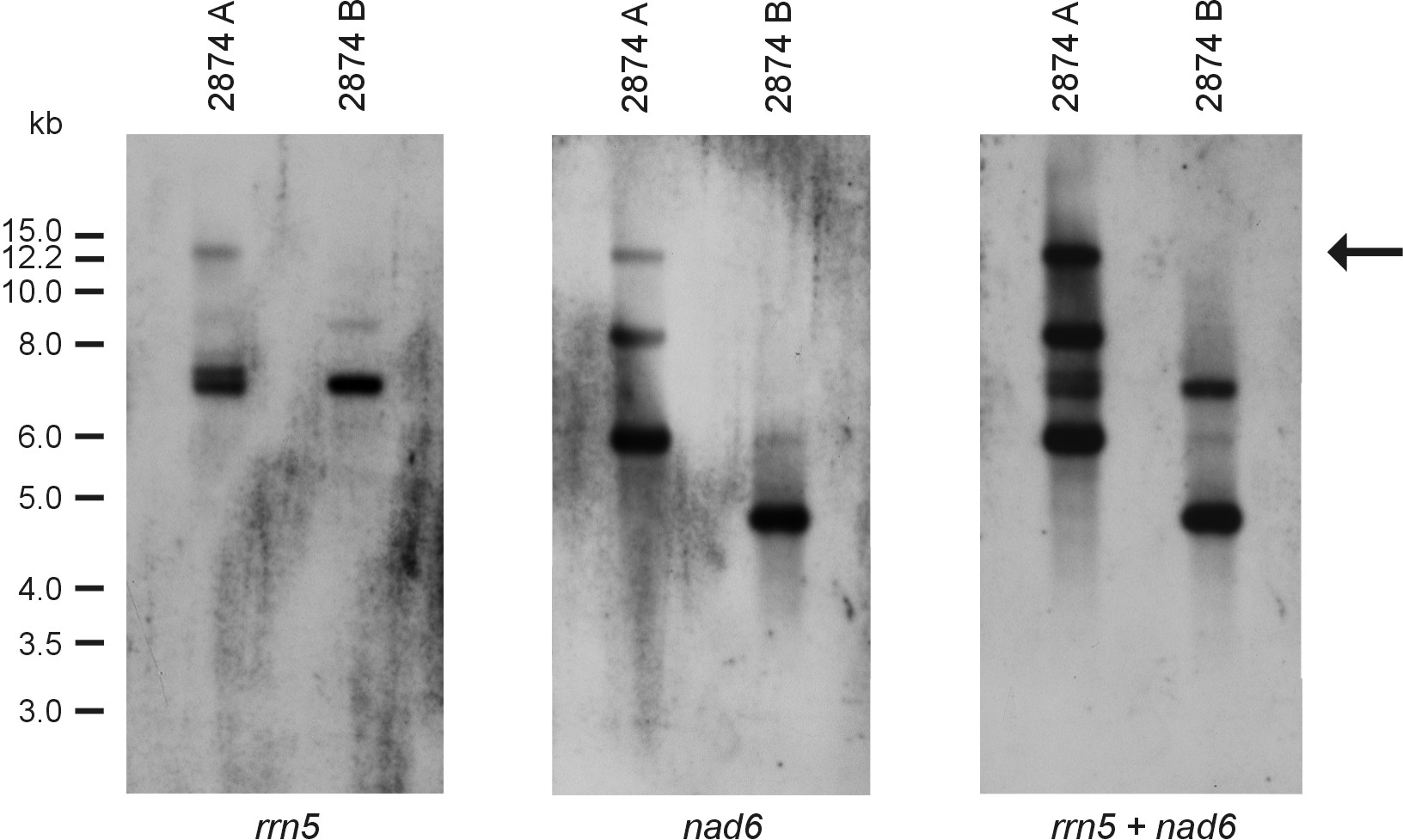


Fig. S3. Southern blotting of *Bam*HI-digested mtDNAs from lines 2874A and 2874B hybridized to probes representing genes *rrn5*, *nad6* as well as jointly *rrn5* and *nad6*. The arrow indicates the fragment hybridizing to both probes.
